# Towards Auditory Profile-Based Hearing-Aid Fitting: Fitting Rationale and Pilot Evaluation

**DOI:** 10.3390/audiolres11010002

**Published:** 2021-01-16

**Authors:** Raul Sanchez-Lopez, Michal Fereczkowski, Sébastien Santurette, Torsten Dau, Tobias Neher

**Affiliations:** 1 Hearing Systems Section, Department of Health Technology, Technical University of Denmark, DK-2800 Kgs. Lyngby, Denmark; mfer@health.sdu.dk (M.F.); sesn@oticon.com (S.S.); tdau@dtu.dk (T.D.); 2 Institute of Clinical Research, Faculty of Health Sciences, University of Southern Denmark, DK-5230 Odense, Denmark; 3 Research Unit for ORL - Head & Neck Surgery and Audiology, Odense University Hospital, Odense, Denmark; University of Southern Denmark, DK-5000 Odense, Denmark; 4 Centre for Applied Audiology Research, Oticon A/S, DK-2765 Smørum, Denmark

**Keywords:** audiology, hearing-aid fitting, hearing-aid settings, hearing loss compensation

## Abstract

*Background*—The clinical characterization of hearing deficits for hearing-aid fitting purposes is typically based on the pure-tone audiogram only. In a previous study, a group of hearing-impaired listeners completed a comprehensive test battery that was designed to tap into different dimensions of hearing abilities. A data-driven analysis of the data yielded four clinically relevant patient sub-populations or “auditory profiles”. The purpose of the current study was to propose and pilot-test profile-based hearing-aid settings in order to explore their potential for providing more targeted hearing-aid treatment. *Methods*—Four candidate hearing-aid settings were developed and evaluated by a subset of the participants tested previously. The evaluation consisted of multi-comparison preference ratings that were carried out in realistic sound scenarios. *Results*—Listeners belonging to the different auditory profiles showed different patterns of preference for the tested hearing-aid settings that were largely consistent with the expectations. *Conclusions*—The results of this pilot evaluation support further investigations into stratified, profile-based hearing-aid fitting with wearable hearing aids.

## 1. Introduction

Hearing loss is typically treated with hearing aids (HAs). The primary purpose of HAs is to provide gain to the input signal to compensate for reduced audibility. In addition, modern HAs incorporate advanced signal processing algorithms for signal-to-noise ratio (SNR) improvement [1]. Consequently, numerous parameters need to be adjusted as a part of the HA fitting process.

In current clinical practice, the assessment of the hearing deficits of a patient mainly relies on pure-tone audiometry. Based on a fitting rule that typically uses the audiogram of the patient as the only information, the HA amplification is then adjusted. For example, the “National Acoustic Laboratories—Nonlinear 2” fitting rule (NAL-NL2; [2]) is commonly used. This rule relies on a combination of empirical knowledge and modelling that is aimed at maximizing the effective audibility of the speech signal. While NAL-NL2 can be expected to provide a reasonable overall solution, there are also patients whose hearing difficulties are not captured by the audiogram and who may, therefore, benefit from other fitting strategies [3,4,5]. Such fitting strategies could include the adjustment of advanced HA features, which are not yet incorporated into existing fitting rules. For example, noise reduction and directional processing are currently activated based on “life-style” considerations, rather than audiological factors. Although advanced HA features can improve the SNR, the individual preference for these settings differs substantially across listeners, possibly because of unwanted speech distortions that are also typically introduced by these algorithms [6]. Therefore, it is possible that the individualized adjustment of SNR improvement algorithms could improve HA outcome, for example, for patients with poor speech intelligibility in challenging environments.

In a recent study, we identified four clinically relevant subgroups of hearing-impaired (HI) listeners while using a data-driven approach [7]. The listeners were characterized by their degree of perceptual deficits or “distortions”, which were estimated while using a battery of auditory measures tapping into loudness and speech perception, binaural processing abilities, and spectro-temporal resolution [8]. Four archetypal patterns of perceptual deficits—referred to as “auditory profiles”—were uncovered. These profiles vary along two primary dimensions, or types, of deficits: speech intelligibility (SI) and loudness perception (LP) related deficits.

In the medical field, personalized treatments aim at providing tailored solutions to clinically relevant subgroups of patients [9]. Here, a profile-based fitting strategy, including a number of candidate hearing-aid settings (HAS), was proposed and pilot-tested. Listeners with a high degree of LP-related deficits (Profiles C and D) were expected to prefer a gain prescription aimed at loudness normalization [4], while listeners with a high degree of SI-related deficits (Profiles B and C) were expected to prefer HAS with SNR improvement. As such, the present study examined the validity of auditory profile-based HA fitting in terms of subjective preference. A multi-comparison evaluation was performed with a group of participants who had previously been classified into the four auditory profiles. This made it possible to explore whether listeners that belong to different auditory profiles would exhibit different patterns of HA outcome.

## 2. Auditory Profile-Based Fitting Rationale

For profile-based HA fitting, a fitting rule combining the prescription of insertion gain and SNR improvement required for each profile is proposed here. This fitting rule was named Better hEAring Rehabilitation-Level-Frequency-Profile fitting rule (BEAR-LFP), and it can be defined, as follows:

(1) HAS(l,f,p)=0.31HTL(f)+α(l,f,p)+δ(p)

where HTL(f) reflects the hearing threshold in dB hearing level (HL) at the different test frequencies, α(l,f,p) represents the gain correction factors applied for different levels (l) and frequencies (f) for a given auditory profile (p), and δ(p) denotes the SNR improvement that is prescribed for that profile.

**Figure 1:**
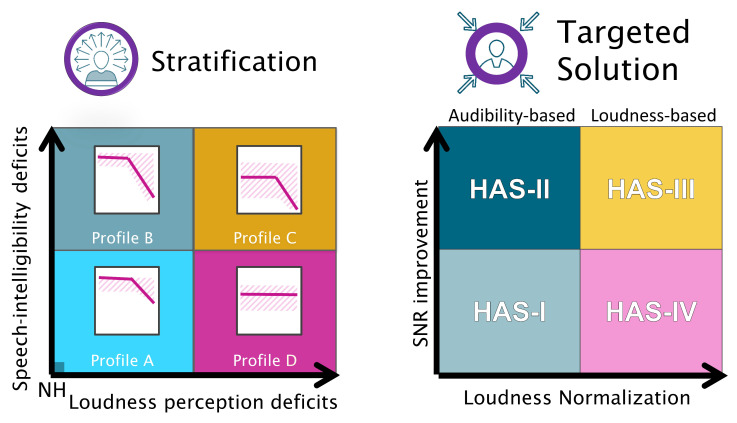
Illustration of the profile-based HA fitting strategy. Left: summary of the results of the auditory profiling [7]. In a two-dimensional space with speech intelligibility-related (SI) deficits on one axis and loudness perception-related (LP) deficits on the other axis, listeners differing in the degree of the two types of perceptual deficits are placed at different positions along the two dimensions. While Profile C represents a high degree of both types of deficits, Profiles B and D reflect hearing deficits dominated by one deficit type only. Profile A has a low degree of deficits and, thus, near-normal hearing abilities. Each dimension covaries with specific deficits observed on a number of behavioral tasks that define a given auditory profile. Right: proposed candidate hearing-aid settings (HAS) for the different profiles, which are intended to compensate for the specific auditory deficits. Signal-to-noise ratio (SNR) improvement as a hearing solution for SI deficits and loudness normalization as a solution for LP deficits.

Figure 1 depicts the proposed stratification (left panels) in the four auditory profiles as a summary of Sanchez-Lopez et al. [7] and the proposed targeted solution (right panels) associated with each of the auditory profiles. Profile A corresponds to a gently sloping high-frequency hearing loss (${\mathrm{HTL}}_{\mathrm{HF}}$ < 50 dB HL), with only mild or no SI and LP deficits. The targeted solution for Profile A is HAS-I, which corresponds to a gain prescription that is based on audibility maximization [10] and priority for good sound quality. Profile B corresponds to a high-frequency sloping hearing loss (${\mathrm{HTL}}_{\mathrm{HF}}$ > 50 dB HL) with SI and temporal resolution deficits. The targeted solution for Profile B is HAS-II, which corresponds to a gain prescription that is based on audibility maximization and SNR improvement. In HAS-II, the use of fast-acting compression and noise reduction might compromise sound quality, but, nonetheless, this solution is expected to provide the most benefit for listeners that belong to Profile B. This is in agreement with the findings of [11], where fast-acting compression provided a systematic audibility benefit. Profile C corresponds to audiometric thresholds >30 dB HL at low frequencies and >50 dB HL at high frequencies, and with SI, LP, spectro-temporal resolution, and binaural-processing deficits. The targeted solution for Profile C is HAS-III, which corresponds to a gain prescription based on loudness normalization [4] and SNR improvement. Because SI is a priority for this profile, aggressive noise reduction and directionality settings might be beneficial for Profile C, even if this compromises sound quality and spatial hearing. Profile D corresponds to a nearly flat hearing loss with audiometric thresholds > 30 dB HL across frequencies, with the only supra-threshold auditory deficit being loudness ecrruitment. The targeted solution for Profile D is HAS-IV, with a gain prescription that is based on loudness normalization and no SNR improvement.

## 3. Pilot Evaluation

For the pilot experiment, a hearing-aid simulator (HASIM) was used, which consisted of three stages: a beamforming stage, a noise reduction stage, and an amplitude compression stage. The beamformer and noise reduction settings were selected based on the achievable SNR improvement [12]. Four candidate HA settings were implemented and evaluated in a multi-comparison experiment.

### 3.1. Candidate Hearing-Aid Settings

The pilot evaluation of the BEAR-LFP rationale was based on four candidate HAS (HAS-I, HAS-II, HAS-III, and HAS-IV). These four HAS were evaluated together with a standard clinical HA fitting (HAS-O). In HAS-I and HAS-II, fast-acting compression was applied in order to provide non-linear gain according to an audibility-based prescription formula. In HAS-III and HAS-IV, slow-acting compression was applied that was based on the principle of loudness normalization. Furthermore, in HAS-II and HAS-III, advanced HA features were activated in order to provide around 2.5 dB of SNR improvement under noisy conditions.

The gain prescription that was used in the current study was derived from an empirical comparison of two existing gain prescriptions: that according to NAL-NL2 [2]; and one other which applies loudness normalization [4]. The individual insertion gain was prescribed while using the one-third gain rule, which was made profile-specific by means of gain correction factors in accordance with LP deficits. In this realization of the BEAR-LFP, a parameter β was used for setting the gain corrections at low, medium, and high input levels that are based on the average differences between the one-third rule. Furthermore, an estimate of the likely loudness summation in profiles C and D was applied for the high input levels by including an additional attenuation for all frequency channels. This is reflected in a gain correction factor $\beta_{HAS}\left(l,f\right)$ that substitutes the parameters α(l,f,p) and δ(p) of expression (1). Because the experiment was a multi-comparison, each participant evaluated all of the HAS candidates, regardless of their specific auditory profile. The four hearing-aid settings tested here (HAS-I to HAS-IV) were implemented, as shown in Table 1.

Table 2 shows the specific settings applied in the HASIM. In order to quantify the effects of the HA signal processing, the HASIM was evaluated in terms of SNR improvement, temporal distortions, and spectral distortions. A head-and-torso simulator (HATS) was placed in the middle of an anechoic chamber with two HA satellites placed behind the ears. The HATS was located in the center of a ring of 24-loudspeakers distributed in the horizontal plane. For the current pilot evaluation, the SNR improvement was achieved while using a cardioid polar pattern in combination with a 9 dB noise reduction, as stipulated in HAS-II and HAS-III. Together, these correspond to a 2.5 dB SNR improvement in a complex scenario containing 24-talker babble [12].

### 3.2. Participants

The recruited participants were stereotypical individuals of their respective auditory profile. Seven listeners participated in the current study (N = 2 in each subgroup except for Profile B, N = 1). All of the listeners had previously completed a comprehensive auditory test battery [8], based on which they were classified [7]. The Science-Ethics Committee for the Capital Region of Denmark approved the study (H-16036391).

### 3.3. Experimental Setup and Procedure

Nine sound scenarios were tested. In each scenario, a fragment of a realistic conversation taken from a publicly available database [13] was used for engaging the listener in the sound scene. The participant was instructed to listen actively to the conversation were two talkers are having a conversation about the differences between two pictures [14]. The tested sound scenarios differed in terms of the background noise. Three noise conditions were included: (1) cafeteria noise (input level 65 dB SPL), (2) traffic noise (input level 75 dB SPL), and (3) quiet. Furthermore, there were three SNR or level conditions. In the case of the cafeteria and traffic scenarios, the target was scaled in level in order to achieve SNRs of −4, 0 or +4 dB. In quiet, the target input level was either 55, 65 or 75 dB SPL. The noise signals were recorded from the microphones of the HA satellites placed on a HATS in either a crowded cafeteria or in a busy street. Impulse responses in the empty cafeteria were obtained while using loudspeakers positioned at azimuth angles of 0° or 90° and simulating the position of two talkers around a table. The impulse responses were convolved with the conversation and noise recordings, and then mixed to create the input signals for the HASIM.

**Figure 2:**
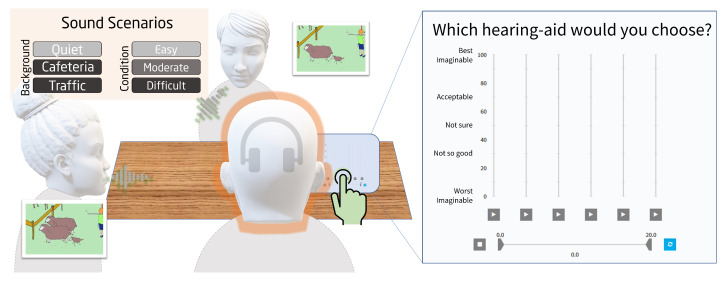
Illustration of the multi-comparison assessment. Left: sketch of the used sound scenarios. The listeners were engaged in a simulated scene consisting of a dialog between two talkers in a quiet or noisy environment. The conversation revolved around finding differences between two Diapix figures [14]. Three sound scenarios and three speech levels or SNRs were used. Right: graphical user interface of the SenseLabOnline software. The four candidate settings were tested in a multi-comparison paradigm that also included a hidden anchor and reference setting (HAS-O). The sound corresponding to a given setting was played back in a loop when the corresponding play button was pressed. The preference judgements were provided using the sliders. The listeners were instructed to (1) identify the anchor, (2) provide a first set of coarse preference ratings between “Acceptable” and “Not good”, (3) reorganize their ratings using the built-in ranking functionality in the SenseLabOnline software [15], and (4) listen to all stimuli again and refine their ratings before storing the final judgements. For each sound scenario, three repetitions per listener were made.

The multi-comparison procedure was realized while using the SenselabOnline software [15]. On a given trial, six stimuli were presented to the listener: an anchor resembling a ‘broken’ HA, a simulated ‘commercial’ HAS as reference (HAS-O), and the four candidate HAS (I, II, III, and IV). The multi-comparisons were performed sequentially across several trials. In each case, a 20-s audio file that corresponds to a given sound scenario that had been processed using the HASIM was played back (Figure 2). The participant then used a slider that ranged from 0 to 100 to rate the sound of each HAS using a Labelled Hedonic Scale [16]. The question posed to the listeners was “which hearing aid would you choose?”. When giving their ratings, they were instructed to focus on their overall preference rather than on specific attributes such as noise annoyance or speech clarity.

## 4. Experimental Results

**Figure 3:**
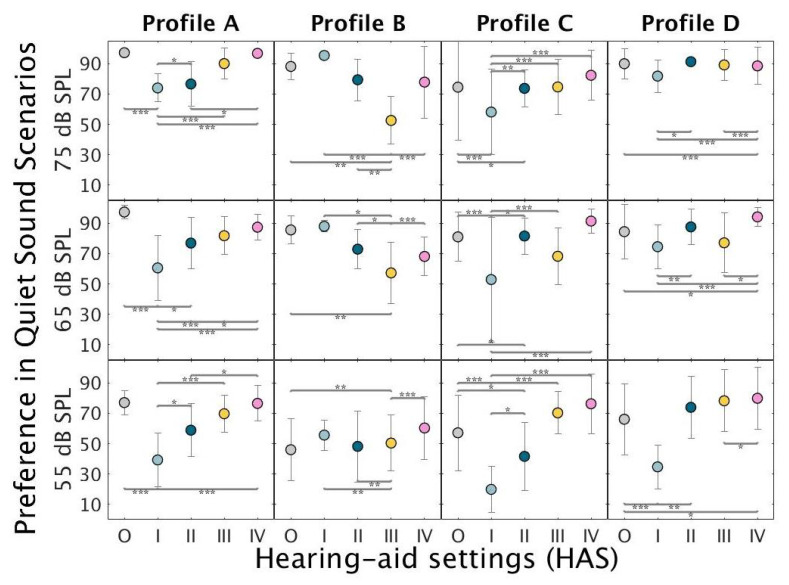
Mean preference ratings for the evaluated HAS (O-IV) in the “Quiet” sound environment across three level conditions: 55 dB SPL (bottom panels), 65 dB SPL (middle panels) and 75 dB SPL (top panels). The circles represent the mean of the scores of the three repetitions performed by the listeners belonging to profile A (**left**), B (**mid-left**), C (**mid-right**), and D (**right**). Error bars show ±1 standard deviation. Significant differences according to a linear mixed model (Appendix A) analysis followed by Tukey’s honest significant differences tests are marked by asterisks. (***) *p* < 0.0001, (**) *p* < 0.001, (*) *p* < 0.01.

Figure 3 shows the mean preference ratings for profiles A, B, C, and D under quiet conditions. Profile-A listeners preferred HAS-O over the two HAS with fast-acting compression across a range of presentation levels (55, 65, and 75 dB sound pressure level, SPL). Profile-B listener preferred HAS-I over HAS-III at 65 and 75 dB SPL; at the low input level (55 dB SPL), they provided the highest rating to HAS-IV. Profile-C and -D listeners showed a preference for HAS-IV and consistently disliked HAS-I.

**Figure 4:**
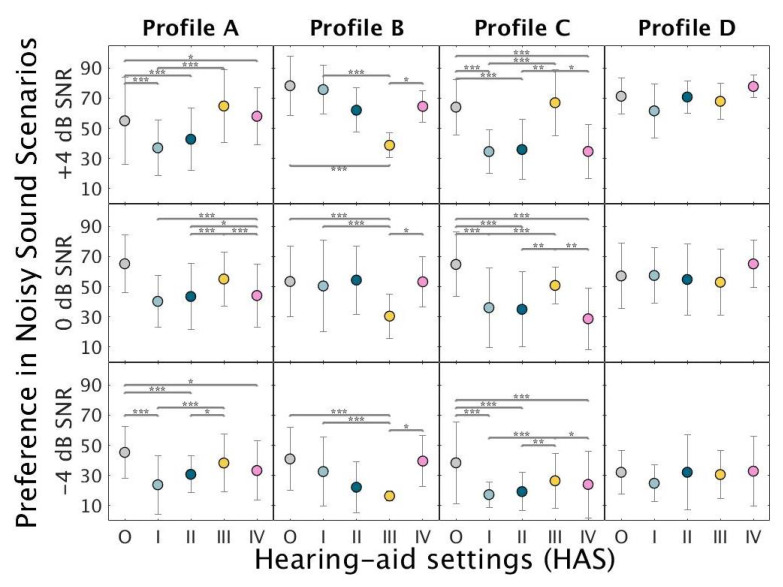
Mean preference ratings for the evaluated HAS (O-IV) under noisy conditions across the three SNR conditions: −4 dB SNR (bottom panels), 0 dB SNR (middle panels) and +4 dB SNR (top panels). Each circle represents the mean of the scores of the three repetitions performed in each noisy sound scenario (traffic and cafeteria) by the listeners belonging to profile A (**left**), B (**mid-left**), C (**mid-right**), and D (**right**). Error bars show ±1 standard deviation. Significant differences according to a linear mixed model (Appendix A) analysis, followed by Tukey’s honest significant differences tests are marked by asterisks. (***) *p* < 0.0001, (**) *p* < 0.001, (*) *p* < 0.01.

Figure 4 shows the mean preference ratings under noisy conditions. Profile-A listeners preferred HAS-III and HAS-O over HAS-I. Profile-B listener consistently disliked HAS-III and showed a preference for HAS-O, HAS-I, and HAS-II. Profile-C listeners preferred HAS-III over the other HAS at higher SNRs (0 and +4 dB). However, HAS-O was also preferred at lower SNRs. Profile-D listeners only showed significant differences at +4 dB SNR, with HAS-IV receiving the highest ratings and HAS-I the lowest ratings.

## 5. Discussion

The current study proposed a fitting rationale for more individualized HA fitting and explored patterns of HAS preference in listeners that belonged to four distinct auditory profiles. The results suggest that the Profile-A and Profile-C listeners based their judgements on similar criteria, especially under noisy conditions. In contrast, the Profile-B and Profile-D listeners showed significantly different patterns. While the Profile-B listener disliked the HAS with loudness-based gain prescription and SNR improvement, the Profile-D listeners favored loudness-based gain prescription and showed no preference for SNR improvement. The results that were obtained for the quiet condition support the use of loudness-based gain prescriptions for profiles with a high degree of LP-related deficits. In contrast, SNR improvement was only preferred by one of the two profiles with a high degree of SI-related deficits when tested at positive SNRs (Profile C). The Profile-B listener showed a preference for fast-acting compression, as consistent with previous research [17,18].

In rehabilitative audiology, the patients are stratified depending on their type and degree of hearing loss. For example, bone-anchored devices are only only prescribed for conductive hearing losses and cochlear implants for severe to profound sensorineural hearing loss. Assuming that listeners belonging to the four auditory profiles are candidates for bilateral hearing aids, further considerations should be made in terms of the gain prescription, the advanced features, and the acoustic coupling of real HAs.

### 5.1. Gain Prescription

The gain prescription could be prescribed by different formulas in different sub-populations based on their hearing deficits. Here, the proposed fitting formula applied different correction factors to an audiogram-based nonlinear gain prescription (Expression (1)). This simple strategy allowed for gain adjustments that prioritized either audibility maximization or loudness normalization based on comparisons with other gain prescriptions (Table 1). However, current fitting formulas, for example, NAL-NL2 [2], prescribe the HA gain that is based on an optimization process and a trade-off between two models: a speech intelligibility model and a loudness model. Besides, it considers gender differences in terms of amplification preference, binaural summation, and HA user experience in the gain prescription. Although the complexity of NAL-NL’s prescription rule might appear more individualized, it does not directly consider the listener’s supra-threshold hearing abilities in the models nor measures beyond the pure-tone audiometry.

The results of the pilot evaluation suggested that listeners in Profiles C and D may benefit from a prescription aiming for loudness normalization in opposition to NAL-NL2, which is based on audibility maximization. However, the approach of the NAL-NL2 fitting formula may be revised to allow for a more personalized HA fitting. Regarding the findings from [7] and the present study, the models used in the NAL-NL2 prescription may be modified to include SI-related deficits and LP-related deficits as input parameters. This can be implemented in the clinic by using the results of loudness scaling tests that are needed for loudness restoration [4] and speech-in-noise perception tests. Consequently, the prescription would apply different criteria, depending on the auditory profile of the listener, and the optimization process would provide a weighted solution where either speech audibility or loudness are prioritized.

### 5.2. Advanced Features

Advanced HA features are useful for providing listening comfort and increasing the satisfaction of the listener in complex situations. Often, the HA features are modified, depending on the sound scene, providing an optimized set of parameters for specific sound environments [19]. It can be argued that current HA technology focuses on the patient’s ecology, such that the HA parameters are automatically modified, depending on the listening condition. For example, SNR improvement algorithms are often activated in challenging situations to improve listening comfort, and deactivated in less challenging situations in order to maintain good sound quality. However, the individualization of the HA parameters based on the hearing deficits might provide an additional benefit. In the present study, different HA settings were tested in realistic scenarios. The listeners with a higher degree of perceptual distortions showed a preference for HA settings with SNR improvement. Importantly, this preference became stronger in scenarios with positive SNRs, i.e., in less challenging sound scenarios. This finding suggests that aggressive parameters of SNR improvement algorithms might still be relevant in more favorable conditions, which is in line with other findings [20], especially in listeners with reduced speech intelligibility in noise.

Advanced HA features are beneficial for noise reduction and listening comfort. However, these algorithms also provide audible distortions. For example, aggressive single-channel noise reduction leads to “musical noise” [21], whereas aggressive beamforming can produce distortions of binaural cues [6]. Wu et al. [22] investigated the speech-in-noise performance of listeners that were divided into the four auditory profiles, who had also participated in a previous auditory profiling study [7]. All of the listeners experienced an improvement when the target speech was in front, and aggressive noise reduction and beamforming were applied. In contrast, speech intelligibility dropped to 0% when the target was presented from one side. These findings suggest that the trade-off between SNR improvement and audible distortions introduced by the HAs might be guided by individual hearing deficits. Listeners with larger difficulties in terms of speech understanding might profit from more aggressive parameters. However, hearing tests fpr evaluating the acceptable limits of these aggressive parameters might be necessary in future audiological practice.

### 5.3. Acoustic Coupling

In real HAs, the acoustic coupling can compromise the targeted insertion gain and noise reduction. Ear-moulds with large vents or instant eartips affect the effective amplification at low frequencies, the effective noise reduction [23], and increase the risk of acoustic feedback [24]. In the present study, listeners from Profiles C and D showed a preference for settings with low-frequency amplification, while listeners from Profiles B and C indicated a preference for SNR improvement. These findings suggest that closed fittings are more adequate for listeners from Profiles B, C, and D. However, other burdens are typically associated with occluding fittings [25], such as the abnormal perception of the listener’s own voice. This should be taken into account in the evaluation of this approach with real HAs, and the acoustic coupling should be carefully evaluated by, for example, real-ear measurements.

## 6. Conclusions

The present study proposed an auditory profile-based fitting rationale for more stratified HA treatment. The results of a pilot evaluation showed small, but significant, differences in terms of preferred HA setting among listeners that belonged to four distinct profiles. Overall, these initial findings provide a useful basis for further investigations into profile-based HA fitting, which will include field trials with wearable devices and objective assessments, such as speech intelligibility tests.

## Figures and Tables

**Table 1:** Gain prescription for the four candidate hearing-aid settings (HAS). Non-linear gain was calculated for 50, 65, and 80 dB SPL. The gain was calculated based on the hearing thesholds (HTL) and the HAS tested. For each HAS, a correction factor $\beta_{HAS}$ was applied that reflected different gain prescription approaches (i.e., audibility maximization [10] and loudness normalization [4]).

Insertion Gain $=0.31\mathrm{HTL}\left(f\right)+\beta_{\mathrm{HAS}}\left(l,f\right)$						
HAS-I $\beta_{I}\left(l,f\right)$	250 Hz	500 Hz	1 kHz	2 kHz	4 kHz	>6 kHz
Target 50	-	-	+ 3	+ 7	+ 7	+ 5
Target 65	-	-	−2	0	0	0
Target 80	-	-	−5	−5	−5	−5
HAS-II $\beta_{II}\left(l,f\right)$	250 Hz	500 Hz	1 kHz	2 kHz	4 kHz	>6 kHz
Target 50	−3	−3	+ 3	+ 7	+ 7	+ 10
Target 65	−3	−3	−2	0	0	0
Target 80	−6	−6	−9	−9	−9	−9
HAS-III $\beta_{III}\left(l,f\right)$	250 Hz	500 Hz	1 kHz	2 kHz	4 kHz	>6 kHz
Target 50	+ 2	+ 3	+ 4	+ 6	+ 10	+ 10
Target 65	−10	−10	−5	0	0	0
Target 80	−14	−14	−14	−14	−14	−14
HAS-IV $\beta_{IV}\left(l,f\right)$	250 Hz	500 Hz	1 kHz	2 kHz	4 kHz	>6 kHz
Target 50	+ 2	+ 3	+ 4	+ 6	+ 5	+ 5
Target 65	−6	−6	−6	−3	−3	−3
Target 80	−10	−10	−10	−10	−14	−14

**Table 2:** Hearing-aid settings (HAS) evaluated in the multi-comparison experiment. The directionality (DIR) setting could be either omnidirectional (omni) or a fixed forward-facing cardioid setting. The noise reduction (NR) could provide an attenuation of 5, 9, or 15 dB, following the estimation of the speech signal. For the anchor stimulus, errors were deliberately introduced into the speech signal estimation to achieve a poor sound quality. The attack and release times of the amplitude compressor were similar to those used in previous studies. The HAS were characterized in terms of SNR improvement and spectral and temporal signal distortion in a complex noisy environment (Sanchez-Lopez et al., 2018).

HAS	Anchor	HAS-O	HAS-I	HAS-II	HAS-III	HAS-IV
DIR setting	Omni	Cardioid	Omni	Cardioid	Cardioid	Omni
NR (dB)	15 *	5	Off	9	9	Off
Attack time (ms)	5	250	5	5	5	5
Release time (ms)	10	1250	40	40	1250	1250
SNR improvement (dB)	0	2	0	2.5	2.5	0

* (errors artificially introduced).

## Data Availability

The Data, materials and methods are available at Zenodo. http://doi.org/10.5281/zenodo.3732319.

## Abbreviations

The following abbreviations are used in this manuscript:

BEAR-LFP	Better hEAring Rehabilitation - Level-Frequency-Profile fitting rule
DIR	Directionality
HA	Hearing aid
HAS	Hearing-aid settings
HASIM	Hearing-aid simulator
HL	Hearing Level
HTL	Hearing thresholds
LP	Loudness perception
NAL-NL2	National Acoustic Laboratories—Nonlinear version 2
NR	Noise reduction
SI	Speech intelligibility
SNR	Signal-to-noise ratio
SPL	Sound pressure level

## Appendix A. Linear Mixed Model

The data was analyzed using a linear mixed model. The full model was reduced using backward elimination of random-effect and fixed-effect terms. The resulting model included:

$$\begin{array}{cc} Rating∼ & Profile+Condition+Background+HAS+\ldots \\ \; & Profile\times HAS+HAS\times Background+e_{participants}, \end{array}$$

where $e_{Subject}$ is the random effect of the participants. Since the Participant and Profile were nested, the random factor is indeed the interaction of both factors.

Table A1 shows the analysis of variance (ANOVA) of the model. The honest significant differences (HSD) shown in Figure 3 and Figure 4 correspond to the post-hoc analysis of the linear mixed model using estimated marginal means.

**Table A1:** Analysis of variance of the linear mixed model used in the data analysis.

**Fixed effects**	**Sum Sq**	**Mean Sq**	**NumDF**	**DenDF**	***F* Value**	**Pr (>F)**
Profile	444.71	148.24	3.00	3.00	0.50	0.7102
Condition	112,063.98	56,031.99	2.00	910.00	187.55	<0.0001
Background	165,740.77	82,870.38	2.00	910.00	277.39	<0.0001
HAS	24,207.18	6051.79	4.00	910.00	20.26	<0.0001
Profile:HAS	31,595.01	2632.92	12.00	910.00	8.81	<0.0001
Background:HAS	11,113.38	1389.17	8.00	910.00	4.65	<0.0001
**Random effects**	**npar**	**logLik**	**AIC**	**LRT**	**Df**	**Pr (>Chisq)**
$e_{Subject}$	33.00	−4033.15	8132.30	136.89	1	<0.0001
